# Bacterial Outer Membrane Vesicles: Research Advances from Biogenesis Mechanisms to Engineered Applications

**DOI:** 10.3390/membranes16060208

**Published:** 2026-06-11

**Authors:** Mengyuan Zhang, Xin Zhao, Mingsheng Tang, Wei Zou

**Affiliations:** 1 School of Public Health, Kunming Medical University, Kunming 650500, China; zmy19971018123@163.com (M.Z.) xinxinxin928@163.com (X.Z.); 2 The Affiliated Mental Health Center, Kunming Medical University, Kunming 650224, China; 3 Infection Control Office, Xi’an Public Health Center (Xi’an Emergency Medical Center), Xi’an 710200, China

**Keywords:** outer membrane vesicles (OMVs), primary models, separation and purification, engineering strategies

## Abstract

Bacterial outer membrane vesicles (OMVs) are spherical structures made up of a double layer, they are each nanostructured (20–300 nm), and they are released from all populations of Gram-negative bacteria. The purpose of this review is to synthesize a comprehensive summary of the current state of knowledge about OMV biogenesis, function in biology, and application to biomedical engineering. Using these three known biogenesis mechanisms as a basis for this review, we discuss the mechanisms of OMV biogenesis that have been described as conserved: (1) disruption of outer membrane–peptidoglycan links. (2) periplasmic stress-driven adaptive release is associated with bilayer lipid asymmetry and the use of signaling molecules. OMVs are considered to be “public goods” for the microbe, allowing for nutrient acquisition, resistance to antibiotics, and the potential for horizontal gene transfer between microbes. OMVs exhibit a different duality at the interface of the pathogen host, where the pathogenic OMV is the delivery vehicle for virulence factors and pathogen-associated molecular patterns (PAMPs) leading to host immune response, while the symbiotic OMV (e.g., those produced by *Bacteroides fragilis* (*Bact. fragilis*)) promote regulatory T cell differentiation and mucosal tolerance. The review also addresses the various techniques currently available to isolate OMVs (e.g., ultracentrifugation and size-exclusion chromatographic techniques) and presents engineered/alloying strategies (e.g., genetic modifications to tolR/msbB and surface functionalization) to enhance the viability, safety, and specificity of OMVs for biomedical delivery. Finally, the review addresses significant obstacles related to standardization, batch variation, and in vivo safety associated with synthetic or personalized therapeutics based on OMVs, providing some recommendations for future research in this area.

## 1. Introduction

Outer membrane vesicles (OMVs) are nanoscale lipid vesicles (20–300 nm in diameter) generated from the outer membrane of Gram-negative bacteria. Virtually all bacterial species studied so far have been reported to produce OMVs, which can contain lipopolysaccharides, outer membrane proteins, soluble periplasmic proteins, and nucleic acids [1,2]. Although OMVs were initially considered to be cellular debris, accumulating evidence indicates that they are actively and specifically produced. Several mechanisms have been proposed to explain OMV formation, including disruption of outer membrane–peptidoglycan linkages, excessive stress that may cause abnormal periplasmic pressure, and coupling between the lipid bilayer and the outer membrane [3]. In microbial communities, OMVs are thought to contribute to nutrient acquisition, influence susceptibility to antibiotics, and facilitate horizontal gene transfer, although the magnitude of these effects may vary across species and conditions. At the host–microbe interface, OMVs can act through multiple routes. Pathogenic OMVs have been reported to deliver virulence factors and pathogen-associated molecular patterns (PAMPs), thereby promoting host immune activation [4]. By contrast, symbiotic OMVs—such as those produced by *Bacteroides fragilis* (*Bact. fragilis*) PSA-OMVs—have been associated with regulatory T cell induction and increased IL-10, supporting mucosal tolerance [5,6]. For isolation and purification, ultracentrifugation, ultrafiltration, and size-exclusion chromatography are commonly used. Given their immunogenic potential, biocompatibility, and amenability to engineering, OMVs have been explored for vaccine development, drug delivery, and diagnostic applications [7]. Engineering bacterial strains to produce OMVs—for example, using TolR protein (tolR) and lipid A myristoyltransferase MsbB (msbB) mutant strains—and modifying OMV surfaces (e.g., PEGylation or scFv conjugation) may improve production efficiency, reduce endotoxicity, and support more targeted delivery [8,9]. Despite these advances, important issues remain unresolved, including standardization across batches, the impact of OMV heterogeneity, in vivo safety, and the feasibility of achieving desired cargo loading. Future directions may include synthetic OMVs and personalized OMV-based vaccines, provided that their function, safety, and manufacturability can be established in relevant models [10].

This review systematically assesses the structure, biology, and function of OMVs, including OMV populations in bacteria and at the interface of the host and bacteria. It covers isolation methods, recent applications in vaccines, drug delivery, engineered modifications, and future challenges.

## 2. Structure and Composition

### 2.1. Basic Structural Characteristics

OMVs are nanoscale, near-spherical structures that bud from the outer membranes of Gram-negative bacteria [11]. Structurally, an OMV can be viewed as a single lipid bilayer containing an aqueous lumen, functioning as a nanoscale extension of the outer membrane.

**Figure 1:**
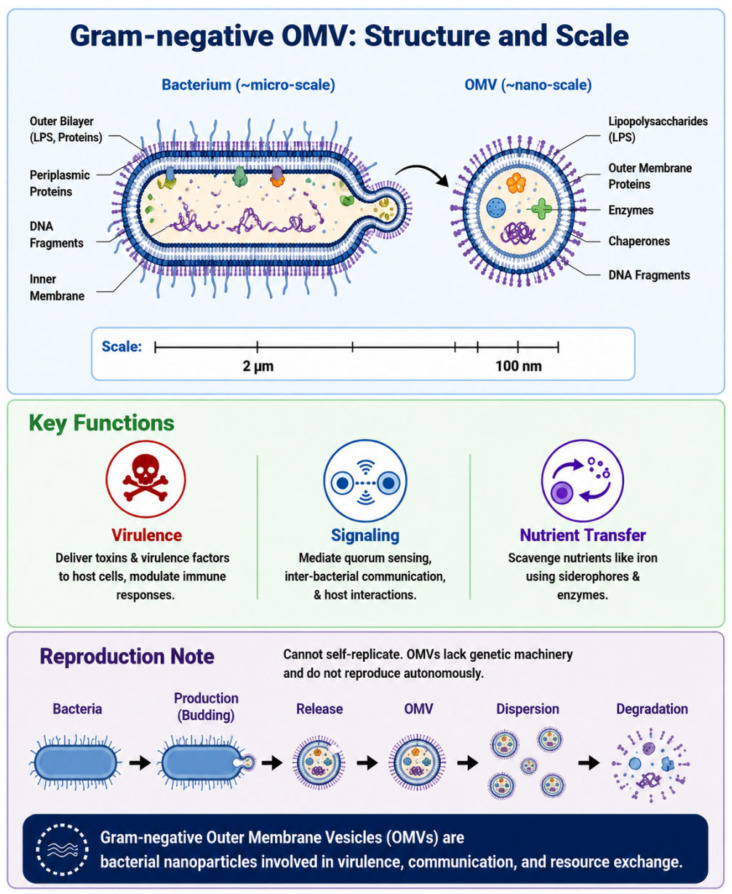
Structure and composition; OMV life cycle illustrates the production, release, dispersal, and degradation of OMVs.

The outer bilayer incorporates lipopolysaccharide (LPS) and phospholipids, and it is enriched in outer membrane proteins, including porins and factors associated with virulence, adhesion, and immune modulation, while largely retaining characteristics of the parental outer membrane [12,13]. The lumen is believed to resemble the periplasm. It therefore harbors periplasmic proteins, hydrolases involved in cell wall remodeling and antibiotic degradation, and small-molecule metabolites [14]. Nucleic acids (for example, fragmented DNA and RNA) have also been detected. However, OMVs do not contain complete, replicable genomic structures. Functionally, they transport bioactive cargoes that can support virulence, signal transduction, nutrient acquisition, and broader microbial interactions [15,16]. Because OMVs lack full genetic material and replication machinery, they cannot self-replicate. Instead, they depend on ongoing bacterial production and turnover, consistent with a cycle of production, release, dispersion, and degradation [17,18,19]. Taken together, OMVs are often described as functionalized extensions of bacteria, linking outer membrane and periplasm-associated functions with the selective delivery of specific components over distance. This structural organization is considered important for bacterial adaptation and pathogenicity (Figure 1) [20].

### 2.2. Lipid Composition

OMV membranes generally resemble the parent outer membrane, but they also show selective sorting and enrichment of lipids. At the biochemical level, the OMV bilayer is composed mainly of phospholipids and LPSs, which together shape its physicochemical properties, membrane curvature, and biological activity. In the inner leaflet, phospholipids such as phosphatidylethanolamine (PE), phosphatidylglycerol (PG), and cardiolipin are commonly reported [18,21]. Among them, PE is often the most abundant lipid. By providing amphiphilic character and supporting appropriate membrane packing, PE is thought to help maintain the fluidity and structural features that favor curvature.

PG and cardiolipin, both negatively charged, can contribute to surface charge and to curvature-associated stresses at the budding region of the outer membrane [22,23]. Consistent with this idea, differences in phospholipid composition and ratios can influence membrane fluidity, thickness, and lipid stacking. These changes may help stabilize OMV morphology. Beyond composition alone, subtle lipid differences between the outer membrane and OMVs have been proposed to support vesicogenesis and outward budding [24]. LPS is another key determinant. The lipid A component contributes to the endotoxic activity of OMVs and can trigger innate immune activation through the TLR4/MD-2 pathway in response to bioactive LPS. In addition, the immunogenicity and host–microbe interactions of OMVs depend not only on the lipid A moiety, but also on the chemical features of the core oligosaccharide and the O-antigen region, which together define the overall LPS chemistry and surface properties [25,26]. Experimental evidence also suggests that LPS species are not incorporated randomly during vesiculation. For instance, OMVs from Pseudomonas aeruginosa reportedly contain predominantly B-band LPS, whereas the outer membrane is enriched for A-band LPS [13,27]. Selective enrichment of B-band LPS in OMVs may have at least two consequences. First, it can influence OMV immunology: higher levels of negatively charged, immunostimulatory LPS may promote stronger innate immune responses, potentially shifting complement activation and PRR recognition [28,29]. Second, accumulation of anionic LPS may change local surface charge and lipid packing, thereby reshaping curvature stress. These biophysical shifts are believed to lower bending energy barriers and favor OMV formation [30]. Overall, specific phospholipid classes and LPS variants help shape OMV membrane structure, with downstream effects on OMV stability and function.

### 2.3. Protein Composition

OMV structure and function depend on proteins, and OMV-enriched proteins are often grouped into three broad categories. First, outer membrane proteins (OMPs)—including OmpA, OmpC, and OmpF porins—are thought to help maintain OMVs as intact, permeable structures by mediating hydrophilic interactions [31]. In OMVs, OMPs can retain native-like conformations and reflect key properties of the parent outer membrane. Second, OMVs contain periplasmic enzymes, such as alkaline phosphatase, proteases, glycosidases, lipases, and enzymes involved in peptidoglycan modification. By sequestering these enzymes within the vesicle, OMVs can shield them from extracellular proteolysis and provide a route for their release into the extracellular environment. This protective compartmentalization may also increase local enzyme concentrations and support enzyme stability relative to their free counterparts, thereby enhancing localized activity [28,32]. Third, bacterial pathogens can package virulence-associated factors—for example, adhesins, toxins, invasion proteins, effector proteins, and factors that promote immune evasion—into and through OMVs. Relative to soluble factors, group delivery from a single vesicle can improve protection from degradation and help coordinate multiple effector molecules, supporting integrated pathogenic processes [33,34]. Consistent with this functional specialization, proteomic analyses indicate that OMV proteins are not simply a diluted copy of outer membrane proteins. Instead, OMV protein profiles are markedly distinct, suggesting that OMVs selectively enrich certain proteins while depleting others, rather than containing parental membrane proteins in proportion to their abundance [32,35].

### 2.4. Nucleic Acid Composition

OMVs have been implicated in mediating nucleic acid transfer, which supports the view that nucleic-acid-associated gene transfer can contribute to intercellular communication. DNA associated with OMVs—whether originating from plasmids or chromosomal DNA—may be present either inside the vesicle or in membrane-associated forms. Encapsulation of DNA within OMVs can help protect it from degradation by nucleases, improve its persistence over time, and increase the chances of uptake by recipient cells [36,37,38]. Once delivered, OMV-associated DNA can, in principle, promote horizontal gene transfer between bacteria. This may enable the spread of traits such as antibiotic resistance, virulence determinants, and metabolic functions, thereby positioning OMVs as an additional route for genetic exchange [39,40]. In addition to DNA, OMVs contain diverse RNA species, including rRNA, tRNA, mRNA, and multiple classes of small RNAs (sRNAs). Some sRNAs have been reported to be enriched in OMVs, consistent with non-random or actively regulated sorting processes [2,41,42]. OMV-associated sRNAs can remain stable in harsh conditions and may act on distant cells after uptake. Upon internalization, these sRNAs have been described to regulate gene expression and to influence host inflammation and immune responses, which could contribute to immune evasion and shaping of a permissive microenvironment [11,15,43]. The evidence suggests that OMVs are more than simple carriers of proteins and lipids. They can be considered multi-component signaling systems in which structural components, enzymes, toxins, signals, and nucleic acids are co-delivered in a coordinated manner [2]. Finally, nucleic acid transfer mediated by OMVs may support functional synergies within microbial communities. During host–pathogen interactions, OMV-delivered DNA and RNA can alter host gene expression and immunological status, adding an additional layer of regulation to host responses. Thus, OMVs can be viewed as multi-functional platforms for cross-talk between cells and tissues through the combined delivery of biochemical effectors and genetic information [44,45].

## 3. Biogenesis Mechanisms (Figure 2)

### 3.1. Physical Constraints and Fundamental Requirements

Across Gram-negative species, OMV formation is increasingly interpreted as the convergence of (i) mechanical decoupling between the outer membrane and peptidoglycan-containing periplasm and (ii) local membrane remodeling that generates the curvature and forces required for neck formation and vesicle scission. Classic studies have shown that disruptions in envelope integrity and periplasmic stress can promote OMV release, while more recent work has highlighted that the lipid environment (e.g., outer membrane composition and asymmetry) can bias membrane bending toward discrete high-curvature domains. Importantly, mechanistic conclusions depend on how OMVs are isolated and characterized, since co-enrichment of periplasmic contaminants or membrane fragments can affect cargo-based inference. Therefore, an integrative view—linking envelope mechanics, periplasmic proteostasis/peptidoglycan perturbations, and lipid-driven bilayer remodeling—provides the most robust framework for interpreting OMV biogenesis [13,18,32].

Interlayer-linking molecules—such as Braun lipoprotein (Lpp), OmpA, and components of the Tol–Pal complex—help couple the layers of the Gram-negative cell envelope. By maintaining these connections, they can stabilize the envelope and reduce disordered shedding of the outer membrane [46,47,48]. Given this coupled architecture, OMV budding is often described as requiring two conditions.

1. Disruption of local linkage between the outer membrane and peptidoglycan. Weakening Lpp–peptidoglycan cross-links and/or reducing OmpA-dependent attachment, and/or perturbing Tol–Pal integrity in specific microdomains, can allow the outer membrane to be displaced. This may generate a “structurally relaxed zone” from which OMVs bud [49,50].

2. Sufficient membrane curvature and shear/contraction forces. Even with a weakened connection, the requirement for outward bending of the outer membrane will be necessary from the altered lipid composition, increasing protein accumulation, and/or development of tensions resulting in an imbalance will create sufficient forces concentrated at the “neck”of the vesicle, resulting in cleavage and detachment [51,52].

**Figure 2:**
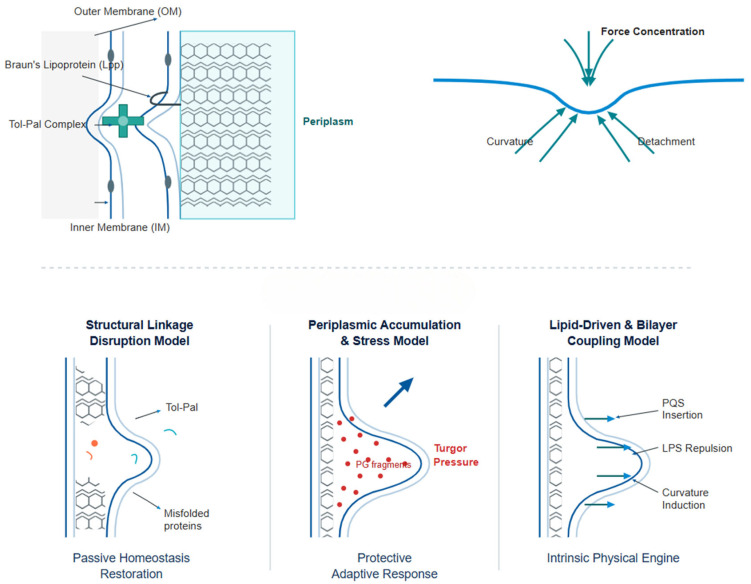
Mechanisms of biogenesis.

### 3.2. Structural Disruption Model

Gram-negative bacteria couple their outer membranes and peptidoglycan layers through linkage proteins, such as Lpp, OmpA, and Tol–Pal, which contribute to the mechanical stability of these structures. The Model of Structural Linkage Disruption proposes that weakened linkages arise from either genetic factors (for example, deletion of Lpp or mutations of OmpA) or environmental conditions (for example, impaired Tol–Pal function). This weakening may uncouple the outer membrane from the peptidoglycan layer, concentrate localized mechanical stress, and promote abnormal membrane curvature. Under these conditions, the outer membrane can bulge and bud outward, which can result in OMV formation [49,53,54]. Generation of OMVs is a passive to quasi-active re-establishment of membrane homeostasis through the release of excess or imbalanced segments caused by the disruption or rearrangement of interlayer connection.

In support of this concept, Bernadac et al. demonstrated that *Escherichia coli* (*E. coli*) Tol–Pal mutants form outer membrane vesicles, consistent with the idea that Tol–Pal contributes to controlling OM stability and organization rather than simply responding to stress [49]. More broadly, Schwechheimer et al. consolidated the view that envelope control mechanisms—including structural constraints imposed by OM envelope systems—govern OMV production [53].

### 3.3. Cytoplasmic Accumulation and Stress Model

The periplasmic accumulation and stress model provides a basis for understanding how OMV biogenesis may contribute to cellular homeostasis. In the periplasm, proteins are positioned for proper folding, modification of peptidoglycan, and related metabolic processes. When cells experience stress—such as antibiotic exposure, oxidative stress, peptidoglycan imbalance, or failures in protein quality control—misfolded proteins, fragments of peptidoglycan, and potentially toxic metabolites may accumulate in the periplasm. These changes can alter osmotic pressure and ionic composition, thereby imposing mechanical stresses on the system [51,55,56]. Consistent with this hypothesis, experimental studies have reported that OMV production rates increase under such stress conditions. They also indicate that OMVs isolated from stressed cells often contain a cargo profile enriched for misfolded proteins and damaged subcomponents, compared with OMVs obtained under steady-state conditions. Together, these observations suggest that OMV production may function as part of an adaptive response. At the same time, the evidence also supports a more protective interpretation: OMVs may help buffer periplasmic stress rather than simply reflecting cellular damage [57].

### 3.4. Lipid-Driven and PQS Bilayer Coupling Model

The OMVs of Pseudomonas aeruginosa are driven by the lipid composition and bilayer asymmetries of the outer membrane in addition to other structural and periplasmic factors. The “Lipid-Driven and PQS Bilayer Coupling Model” is demonstrated in Pseudomonas aeruginosa [58,59].

The Pseudomonas quinolone signal (PQS) that is present in Pseudomonas aeruginosa probably interacts with LPS as it inserts into the outer leaflet (OL) of the OM. The increased concentration of PQS relative to LPS enhances the total amount of lipids in the OL, generating an asymmetrical distribution of areas in the bilayer and transmitting stresses across the bilayer. The presence of these stresses is likely responsible for the bulging outwards of the OM, resulting in the creation of areas of high curvature at which vesicles can be formed. The generation of additional curvature, along with mechanical or protein interactions to the bilayer, results in the release of OMVs [60,61,62]. The localized accumulation of negatively charged LPSs or certain phospholipids has the potential to modify the physical characteristics of the OL so that certain regions have increased propensity to bend. In accordance with the PQS model, the uneven distribution of lipids, the modified ratio of the inner and outer layers, and specific interaction of small molecules or lipids within the lipid bilayer serve as the intrinsic physical “engines” for OMV budding [63,64].

## 4. Biological Functions (I): Inter-Bacterial Interactions (Figure 3)

In bacterial communities, especially in highly populated biofilms, OMVs serve as “public resources” that function in collective survival, adaptation, and evolution [65,66]. OMVs facilitate coordination of population level processes for defense, nutrient acquisition/use, and community remodeling.

**Figure 3:**
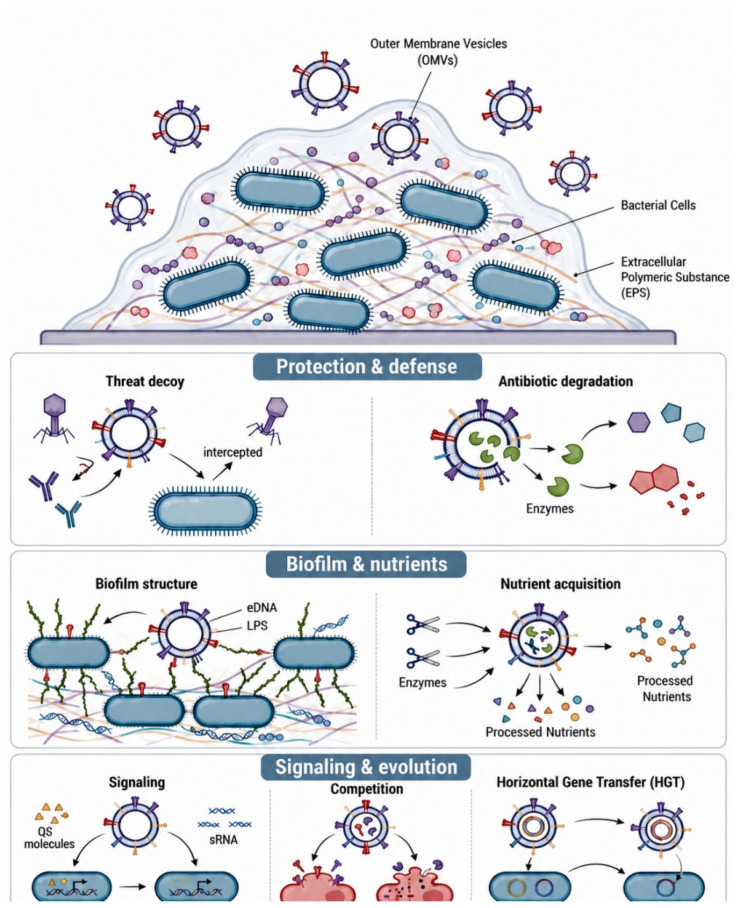
OMVs operate as a “common resource” within biofilms, filling three key functions: collective defense, biofilm construction and sharing of nutrients, and signal transduction/interspecies competition/horizontal transfer of genes.

### 4.1. Public Goods and Collective Defense

OMVs can be described as “public goods”, as they provide both a protective and a metabolic function to the population:

1. OMVs can act as a “decoy” against host or phage attack. Because OMVs display LPSs, membrane proteins, and antigens that are homologous to those on the parent bacteria, they can bind to phages as well as to complements and antibodies. In this way, OMVs can intercept the attacker and reduce how effectively the attacking organism interacts with live bacterial cells [67,68]. For example, OMVs from *Pseudomonas aeruginosa* (*P. aeruginosa*) readily adsorb to phages, lowering the probability of infection. After binding complement or antibodies, OMVs can form a “buffer layer” on biofilms, helping protect internal bacteria from being cleared [69,70].

2. OMVs can also contribute to a “collective protective barrier” by releasing resistance factors. When resistance determinants are packaged in OMVs, they can function at the population level. For instance, OMV-encapsulated β-lactamases can degrade β-lactam antibiotics in the extracellular environment, thereby reducing the local drug concentration [71,72]; this protects the susceptible organisms within the population, thereby promoting collective resistance to the drug [73,74]. Thus, when faced with stress, OMVs become critical chemical buffers and defense mechanisms.

### 4.2. Biofilm Formation and Nutrient Sharing

In biofilms with a defined architecture, OMVs contribute to the extracellular matrix by enabling matrix formation, stabilization, and nutrient utilization.

1. OMVs provide “molecular glue”. Surface polysaccharides on OMVs, together with adhesion proteins and extracellular DNA (eDNA), promote attraction and cell–cell adhesion, thereby enhancing the structural integrity and stability of the biofilm. OMVs can also occupy spaces within the biofilm architecture, increasing biofilm density and mechanical strength. In this context, an eDNA/protein/LPS network can function as a matrix that helps preserve biofilm cohesion under fluid shear forces and/or immune pressure [75,76].

2. Enzymatic degradation of macromolecules and nutrient sharing. OMVs contain diverse extracellular hydrolases, including proteases, lipases, nucleases, and polysaccharide hydrolases. These enzymes can digest complex substrates associated with the outer surface of OMVs and/or within the OMV lumen [77]. Digestion targets include mucins, ECM components, environmental proteins, and eDNA [78]; by breaking down large substrates into smaller metabolites, OMVs generate diffusible products that can leave the vesicle and be used by other members of the biofilm. This supports more efficient overall resource utilization and promotes metabolic sharing within the community. Overall, OMVs are important both for building the matrix and for accessing resources required for biofilm success [79].

### 4.3. Signal Transmission and Interspecies Competition

Besides providing structural support and defense, OMVs can serve important roles in signaling and competition within bacterial populations across different species.

1. Some QS bacteria use OMVs to help reach and maintain quorum. By packaging QS molecules, regulatory proteins, and sRNAs within OMVs, cells can achieve more stable transport and an enhanced effective range/stability of quorum-sensing signals—particularly in Pseudomonas aeruginosa, where PQS can associate with OMVs and diffuse along with them [80]. In addition, gene expression changes in recipient cells driven by OMV-delivered sRNAs and transcription factors provide a basis for “modular” regulation of the population as a whole [81].

2. Toxin- and lysozyme-mediated interspecies competition and community reshaping OMVs can concentrate and deliver aggressive effectors. For example, lysozyme, peptidoglycan hydrolases, and other cell-wall-degrading enzymes can selectively disrupt the cell walls of competing bacteria. In parallel, pore-forming toxins and bacteriocin-like proteins can exert lethal effects [82,83]. Together, this contributes to interspecies competition and reshaping of community composition.

3. OMV-mediated genetic exchange (chromosomal fragments and mobile elements) OMVs can contain chromosomal fragments, plasmid DNA, and/or other mobile genetic elements. DNA carried by OMVs enables ongoing and rapid horizontal transfer of resistance genes, virulence islands, and metabolic islands between bacteria, thereby accelerating adaptive evolution. In this way, nucleic acid transfer mechanisms mediated by OMVs—where vesicles provide protection and diverse cargo combinations—can complement other horizontal transmission routes [84,85,86].

## 5. Biological Functions (II): Dual Role at the Bacteria–Host Interface (Figure 4)

OMVs can support two contrasting outcomes at the bacteria–host interface. Bacteria-derived OMVs that carry virulence factors have been linked to stronger inflammatory signaling in the host, whereas OMVs from non-pathogenic or symbiotic bacteria—for example, members of the gut microbiota—have been reported to contribute to immune tolerance and mucosal homeostasis [87]. These effects are not fixed. The biological outcome appears to depend on the OMV cargo, the local immune state, and the source of the vesicles [88].

**Figure 4:**
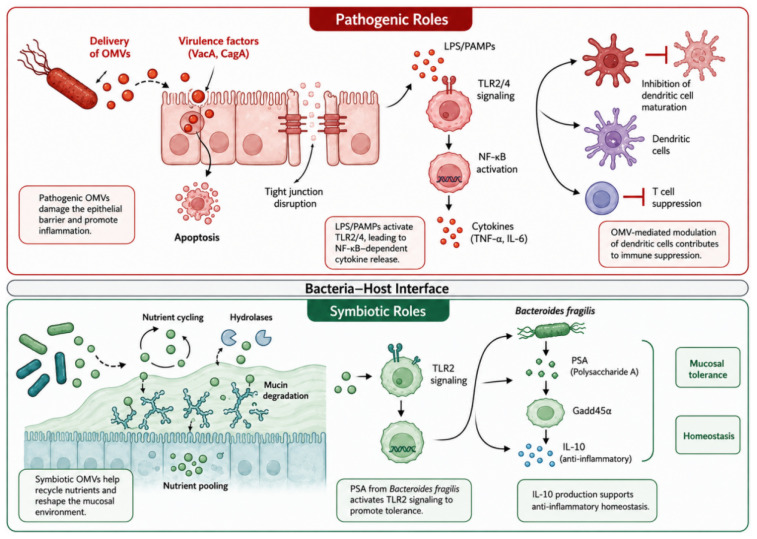
Biological functions (II): dual role at the bacteria–host interface.

### 5.1. Pathogenicity-Related Functions

Gram-negative pathogens utilize OMVs to carry virulence factors over long distances [89]. OMVs also improve the efficacy and efficiency of targeted delivery by protecting the virulence factor from degradation.

1. OMVs penetrate the mucus on the surface mucosal epithelium and come into contact with epithelial cells to deliver virulence factors. For example, OMVs derived from *H. pylori* contain vacuolating cytotoxin A (VacA) and cytotoxin-associated gene A (CagA) that penetrate gastric epithelium to induce gastric injury and damage (apoptosis), disrupt tight junctions, and activate inflammatory signaling pathways, thereby disrupting the mucosal barrier and promoting inflammation [90,91]; OMVs derived from *Porphyromonas gingivalis* (*P. gingivalis*) contain a family of cysteine proteases (gingipains) that play a key role in the degradation of the extracellular matrix, host defenses and immune receptors in periodontal tissues, resulting in the degradation of local architecture and reshaping of the inflammatory response [92].

2. Another pathogenic mechanism relates to immune recognition. OMVs can be enriched in pathogen-associated molecular patterns (PAMPs), including LPS, lipoproteins, peptidoglycans, CpG DNA, and bacterial RNA. These PAMPs can engage pathogen recognition receptors (PRRs), such as Toll-like receptors (TLRs) 2, 4 and 9, and NOD-like receptors, thereby triggering NF-κB, MAPK, and IRF pathway activation. Immune cells may then produce pro-inflammatory cytokines and chemokines, which can initiate and amplify inflammatory responses [93,94,95,96]. At the same time, immune activation does not only reflect defense. These defensive signaling routes can be exploited by bacteria under certain conditions.

3. During chronic infection, pathogens may also use OMVs to dampen host immunity. Some OMVs have been reported to inhibit dendritic cell (DC) maturation by reducing major histocompatibility complex (MHC) and co-stimulatory molecule expression and/or by limiting T cell activation [97,98]; other OMVs can impair T cell proliferation and affect neutrophil chemotaxis and bactericidal activity, which may reduce bacterial clearance by the host [99]. Taken together, pathogens may adjust the amount of OMVs released to reach an equilibrium between “pro-inflammatory” and “immunosuppressive” effects and thus evade the host [100]. This model remains context-dependent and likely varies across bacterial species and infection settings.

### 5.2. Symbiotic-Related Functions

As part of symbiotic microbiota—particularly the gut microbiome—OMVs generally help maintain balance between the microbiome and the host immune system. They also confer multiple benefits by supporting nutrient metabolism, niche construction, and immune education/tolerance.

1. OMVs produced by symbiotic bacteria carry extracellular hydrolases and metabolic proteins that participate in the degradation of mucin (a high-molecular-weight glycoprotein secreted in the intestinal mucosa) and complex carbohydrates present in intestinal mucus. The resulting products are converted into low-molecular-weight nutrients that can be utilized by the host and/or by other symbiotic bacteria [101]; this represents an important mechanism by which OMVs contribute to intestinal microenvironment development and to the diversity and stability of microbial communities, while improving efficiency in microbial resource use. In addition, the enzymatic activity of OMVs supports metabolic mutualism, providing metabolic infrastructure for diverse microbiota cohabitation in the gut [102].

2. OMVs generated by gut symbionts can significantly contribute to the induction of immune tolerance and maintenance of anti-inflammatory homeostasis in the gut. For example, OMVs from Bacteroides fragilis provide polysaccharides [103]. These polysaccharides can be taken up by dendritic cells (DCs) and promote polarization toward a tolerogenic phenotype via TLR2. Bacteroides PSA-OMVs induce immunomodulatory effects and prevent experimental colitis. DC recognition of OMV-associated PSA through TLR2 enhances T cell regulation and promotes anti-inflammatory cytokine production [104]. Moreover, OMV-driven DC signaling requires Gadd45α. PSA-OMV-treated DCs can regulate T cells and suppress inflammation in experimental models, whereas Gadd45α(−/−) DCs fail to modulate T cells or suppress inflammation [105]. Together, these findings indicate that OMV-mediated transfer of symbiotic molecules can prevent disease, highlighting microbiota–mammal communication.

3. Pathogenic inflammation versus tolerance homeostasis. The composition of OMVs from different sources determines whether they promote pathogenic inflammation or support tolerance homeostasis. Pathogenic OMVs tend to be enriched in inflammatory LPS, virulence proteins, and pro-inflammatory PAMPs, leading to strong immune activation and tissue damage [106,107]. In contrast, symbiont-derived OMVs primarily carry immunomodulatory polysaccharides and tolerance-associated signals, which trigger anti-inflammatory pathways (e.g., IL-10) and limit inflammation [108]. Overall, OMVs can be viewed as “packaged immune information parcels.” Their molecular makeup determines whether they drive inflammatory damage or tolerance, providing a rationale for OMV-based vaccines, immunotherapies, and microbiome interventions.

## 6. Isolation and Purification of OMVs [2,37]

OMV research has improved through better preparation and characterization approaches, but standardization, cross-study comparability, and scale-up still pose difficulties. Most OMV preparation workflows start from bacterial culture supernatants. After removing cells and large debris, investigators typically use physical and physicochemical enrichment steps to increase OMV yield and reduce background material.

UC remains widely used for initial enrichment in basic research UF and SEC are increasingly adopted when higher purity and better scalability are priorities. For immunological, clinical, or vaccine-oriented applications, refined purification strategies are often recommended—for example, SEC following UC—to limit non-vesicular contaminants (Table 1).

Filtration of bacterial cultures has been extensively studied and optimized, especially in relation to small-scale filtration. In a continuous chemostat process for *N. meningitidis*, steady-state OMV concentrations reached approximately 4 × 10^11^ OMVs/mL, with reported volumetric productivity up to ~1 × 10^15^ OMVs/L/day when the dilution rate was optimized [113]. This demonstrates the practical feasibility of applying transformations to bacterial outer membrane vesicles.

## 7. Biomedical Applications (Figure 5)

OMVs show broad potential in vaccines, drug delivery, and diagnostics, largely because they are intrinsically immunogenic, possess membrane-like physicochemical properties, and can be engineered for specific functions.

**Figure 5:**
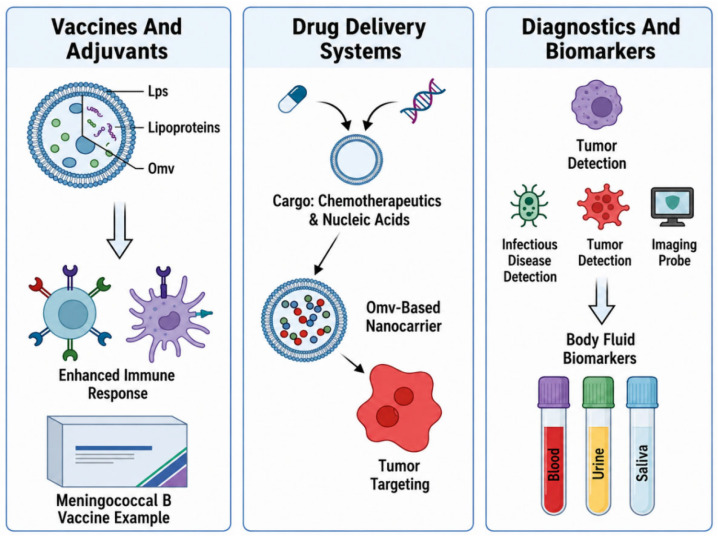
Biomedical applications of bacterial outer membrane vesicles (OMVs).

### 7.1. Vaccines and Adjuvants

OMVs contain LPS, lipoproteins, peptidoglycan fragments, and outer membrane/periplasmic proteins. This composition can provide natural immunogenicity and adjuvant activity, and it may simultaneously support humoral and cellular immune responses [114]. The Meningococcal serogroup B vaccine based on *Neisseriameningitidis* (*N. meningitidis*) OMVs illustrates the translational feasibility of OMV vaccines and supports their safety profile [115,116]. Compared with some conventional adjuvants, OMVs can activate multiple PRRs [117] and may not require exogenous adjuvants. They also have been reported to bias immune responses toward Th1/Th17 and CTL pathways [118,119].

### 7.2. Drug Delivery Systems

Because OMVs are built around a bilayer lipid membrane, they can be compatible with biological environments and remain relatively stable. This may allow them to cross certain biological barriers and act as nanoscale drug carriers [120]. OMV-based delivery has been explored for small-molecule chemotherapeutics [121], nucleic acid drugs/immunomodulators [122] and targeted delivery to tumors, including approaches that combine delivery with immune-related effects [123].

### 7.3. Diagnostics and Biomarkers

OMVs are found in all body fluids (blood, urine, saliva, CSF) where they exist in a stable state, and their makeup reflects the bacterial species from which they are derived, their metabolic state and their virulence, so they could function as good biomarkers and diagnostic carriers. They can be used for diagnosing/the monitoring of infectious diseases, for identifying tumors and as imaging diagnostic platforms. Through the use of engineered OMVs, they will be able to function as visual diagnostic/tracing carriers by incorporating an imaging probe [124] (e.g., use of near-infrared dyes [125], MRI contrast agents [126]). Because OMVs can be manipulated and have shown stability, they may support less invasive biomarker collection and provide a modular basis for diagnostic platform development.

## 8. Engineering Modifications

Engineering at the production strain level optimizes OMV yield, reduces endotoxicity, and enables programmable payload loading.

### 8.1. Engineering Modifications at the Production Strain Level

1. Enhancing OMV production via disruption of the outer membrane–peptidoglycan linker. Bacterial outer membrane–peptidoglycan linker proteins, such as the Tol–Pal system, contribute to cell envelope stability [127]. Genetic disruption or point mutations in components of this linkage, for example knocking out tolR, weaken tethering between envelope layers. This can increase outer membrane blebbing and budding, thereby raising OMV release [128]. Studies in both model strains and pathogenic bacteria support the concept of “high-yield vesicle strains,” suggesting that this genetic lever can provide a practical basis for scalable OMV production.

2. Reducing endotoxicity via reconstruction of the lipid A biosynthetic pathway. Lipid A is the lipid moiety of LPS and is a major driver of strong innate immune activation as well as systemic toxicity [129]. Engineering lipid A biosynthetic genes—including msbB, lpxL, and lpxM—can generate low-toxicity lipid A variants with altered acylation patterns, phosphate content, or acyl chain number [130]. OMVs produced by these engineered strains may preserve adjuvant-like immunostimulatory activity while attenuating excessive inflammatory responses and thereby lowering sepsis-related risk. This rationale supports continued investigation of such OMVs for human vaccine development, systemic administration, and longer-term therapeutic applications.

3. Programmable payload loading: One strategy removes virulence-associated genes and introduces targeted antigen or therapeutic protein sequences into outer membrane/periplasmic proteins. In principle, this supports “selective streamlining” of cargo without disrupting vesicle biogenesis [50,131]. Engineered bacterial strains have been used in vaccine and tumor immunotherapy development to produce “customised OMVs” carrying predetermined combinations of antigens [132].

### 8.2. Structural and Functional Engineering of Vesicles Themselves

At the vesicle level, engineering involves focusing on surface display, physicochemical property regulation, and loading of the cargo.

**Figure 6:**
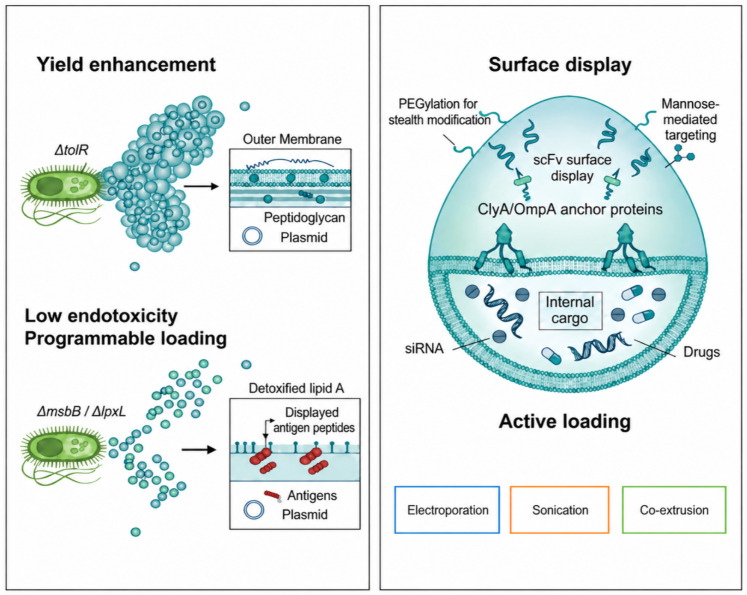
Engineering modifications.

1. Surface Display and Active Target Design: Proteins such as ClyA and OmpA, which anchor in the outer membrane, can act as scaffolds for presenting functional molecules on OMVs. Targeting or immune modulatory cargo can be displayed using, for example, scFv for tumor antigens [133]; mannose to enhance dendritic cell engagement and antigen presentation [134]; and PD-1-related domains/adaptors for immune regulation [135,136,137]. In this way, surface engineering can convert OMVs into targeting carriers with defined receptor recognition.

2. Physicochemical Properties and In Vivo Modulation of the Behavior: To improve pharmacokinetics and reduce rapid clearance by the reticuloendothelial system (RES), surface modifications are commonly applied. PEGylation (covalent or non-covalent PEG modification) can increase hydrophobic–hydrophilic balance and introduce steric shielding, which may reduce plasma protein adsorption and opsonization and thereby prolong circulation [138]. Additional polymer coatings or co-assemblies can also be used to support structural stability and influence OMV release properties.

3. Active Loading and Multifunctional Design of Multiple Payloads: In addition to the natural components of the OMVs, “multifunctional integrated OMV nanodrugs” require efficient, controllable therapeutic loading of the payloads. Some methods of loading the therapeutic payloads into OMVs include: electroporation—high-voltage pulses generate reversible pores, facilitating entry of small molecules and nucleic acids (e.g., siRNA, mRNA, plasmid DNA) into the OMV lumen [139] and sonication—ultrasonic agitation transiently perturbs membranes, improving encapsulation of small and macromolecular cargo and increasing incorporation into OMVs [140]. Repeated extrusion of drug- or nanomaterial-containing OMVs through a defined pore membrane can generate more uniform composite vesicle structures through mechanical shear and mixing [141]. When integrated with gene editing, synthetic biology, and formulation advances, these loading and assembly processes may support translation of OMV platforms from laboratory studies toward clinical evaluation. This could include vaccine development against infectious diseases, tumor immunotherapy, targeted delivery to neoplastic cells, or imaging diagnostics (Figure 6).

## 9. Challenges and Future

Despite progress in OMV research, several critical issues still limit translation. First, OMV production can vary across growth conditions and experimental workflows, which reduces reproducibility. Second, OMVs often remain heterogeneous between batches even when the source strain is the same. Third, the in vivo distribution of OMVs, their safety profile, and the degree of mechanistic understanding are still not fully resolved. Finally, further work is needed to improve cargo loading efficiency and to enhance cargo stability after preparation.

Looking forward, future strategies may combine synthetic biology and nanotechnology to support a more bottom-up, “de novo creation of synthetic OMVs” or OMV-like structures with controllable features. Such approaches could increase yield and reduce batch-to-batch variability, making it easier to standardize OMV preparation and downstream quality control. In parallel, OMV-based vaccines—particularly those incorporating individualized tumor neoantigens—may become increasingly relevant for tumor immunotherapy and precision medicine. Clarifying how proteins and sRNAs are sorted during OMV biogenesis could enable more deliberate cargo design originating from the vesicle-producing step. Taken together, OMVs represent an interdisciplinary platform at the interface of microbiology, immunology, and nanomedicine, and they are likely to remain an active focus for both basic research and clinical translation.

## Figures and Tables

**Table 1:** Isolation and purification of OMVs.

Method	Definition/Principle	Advantages	Limitations
Ultracentrifugation [109], UC	Low-speed centrifugation and filtration remove large particles, followed by ~100,000× *g* ultracentrifugation to precipitate OMVs.	The procedure is relatively simple and suitable for routine laboratory operations. High purity is typically achievable.	However, the equipment is expensive, time-consuming, and relatively inefficient, often requiring subsequent repeated or further purification steps.
Ultrafiltration [110], UF	Membrane filters with specific MWCOs retain OMVs while small molecules permeate, achieving concentration and buffer exchange.	The device is relatively inexpensive, easy to operate, and suitable for large-scale sample processing.	However, the purification yield is typically lower than that achieved through density gradient centrifugation; multiple treatment cycles are often required to attain satisfactory purity, and the removal of membrane fragments or impurities may be insufficient.
Size-exclusion chromatography [111,112], SEC	Porous packing material separates particles by hydrodynamic volume. Larger OMVs elute first; smaller molecules later.	SEC provides mild conditions, maintaining OMV integrity and activity. It effectively removes low-molecular-weight impurities, significantly enhancing purity.	Single-batch volume is limited, often needing pretreatment (e.g., UF concentration). SEC demands higher quality equipment/materials, requiring greater upfront investment than UC.
Density gradient centrifugation	The separation of OMVs with different densities in a gradient significantly.	Enhances purity and enables partial differentiation among OMV subpopulations.	Its drawbacks include more complex operation, greater sensitivity to gradient preparation and centrifugation parameters, as well as higher overall time consumption and cost.
Precipitation	Enrich OMVs using a precipitant.	The method is simple, low-cost, and suitable for large sample sizes.	The drawback is that the purification efficiency is relatively limited, typically requiring subsequent purification steps, and the precipitating agent may alter or impair the biological activity of OMVs.
Immunoaffinity chromatography	The surface antigens of OMVs were captured using specific antibodies.	Capable of achieving high purity and targeted enrichment of specific OMV subpopulations	The drawback lies in the reliance on specific antibodies and their immobilization, which entails high costs, complex procedures, and the need for optimization of elution conditions.
Immobilized metal affinity chromatography	If outer membrane proteins are tagged with a His-tag, they can be selectively enriched using methods such as Ni-NTA.	The operation is relatively straightforward and highly efficient.	Genetic modification of the strain/exosomal membrane protein is required, and metal ions may affect the biological activity of OMVs, necessitating further evaluation.

## Data Availability

No original data was generated for this review article, so data sharing is not applicable.
